# SR-B1, a Key Receptor Involved in the Progression of Cardiovascular Disease: A Perspective from Mice and Human Genetic Studies

**DOI:** 10.3390/biomedicines9060612

**Published:** 2021-05-27

**Authors:** Irene Gracia-Rubio, César Martín, Fernando Civeira, Ana Cenarro

**Affiliations:** 1 Unidad Clínica y de Investigación en Lípidos y Arteriosclerosis, Instituto de Investigación Sanitaria Aragón (IIS Aragón), Hospital Universitario Miguel Servet, 50009 Zaragoza, Spain; civeira@unizar.es (F.C.); ana.cenarro@gmail.com (A.C.); 2 Instituto Biofisika (UPV/EHU, CSIC) y Departamento de Bioquímica y Biología Molecular, Universidad del País Vasco UPB/EHU, 48940 Bilbao, Spain; cesar.martin@ehu.eus; 3 Centro de Investigación Biomédica en Red Cardiovascular (CIBERCV), Instituto Salud Carlos III, 28029 Madrid, Spain; 4 Departamento de Medicina, Psiquiatría y Dermatología, Universidad de Zaragoza, 50009 Zaragoza, Spain; 5 Instituto Aragonés de Ciencias de la Salud (IACS), 50009 Zaragoza, Spain

**Keywords:** Scavenger receptor B class 1, cardiovascular disease, mice and human genetic studies, high-density lipoprotein, low-density lipoprotein

## Abstract

High plasma level of low-density lipoprotein (LDL) is the main driver of the initiation and progression of cardiovascular disease (CVD). Nevertheless, high-density lipoprotein (HDL) is considered an anti-atherogenic lipoprotein due to its role in reverse cholesterol transport and its ability to receive cholesterol that effluxes from macrophages in the artery wall. The scavenger receptor B class type 1 (SR-B1) was identified as the high-affinity HDL receptor, which facilitates the selective uptake of cholesterol ester (CE) into the liver via HDL and is also implicated in the plasma clearance of LDL, very low-density lipoprotein (VLDL) and lipoprotein(a) (Lp(a)). Thus, SR-B1 is a multifunctional receptor that plays a main role in the metabolism of different lipoproteins. The aim of this review is to highlight the association between SR-B1 and CVD risk through mice and human genetic studies.

## 1. Introduction

Cardiovascular disease (CVD) remains the primary cause of mortality and morbidity worldwide [1]. The principal risk factor for developing CVD is relatively high plasma level of low-density lipoprotein cholesterol (LDLc) [2,3,4]. Numerous epidemiological and clinical investigations have revealed that plasma level of high-density lipoprotein cholesterol (HDLc) correlates inversely with the risk of CVD [5,6]. This association has been described by anti-atherogenic capacities of HDL, comprising its role in reverse cholesterol transport (RCT), in which cholesterol from peripheral tissues is transferred to the liver for excretion in bile and its ability to receive cholesterol from macrophages in the artery wall [7,8]. However, Mendelian randomization studies [9,10] and pharmacological interventional studies [11,12] do not support the concept that HDLc directly reduces the risk of CVD [8]. In addition, a retrospective analysis of large epidemiological studies showed that high HDLc concentration is associated with higher risk for CVD [13,14]. These results support the hypothesis that HDL metabolism and functionality is more important than HDLc levels for CVD risk prediction [14]. Acton et al. identified the scavenger receptor B class 1 (SR-B1) as a high-affinity HDL receptor, which facilitates the selective uptake of cholesterol esters (CE) in HDL into the liver [15]. This receptor is also implicated in the plasma clearance of LDL, very low-density lipoprotein (VLDL) and lipoprotein(a) (Lp(a)), lipoproteins with pro-atherogenic properties [16,17,18,19]. Therefore, SR-B1 is involved in cholesterol homeostasis, lipoprotein metabolism and atherosclerosis [4]. In addition, SR-B1 plays a relevant role in HDL-mediated cellular signaling [20], and might play a crucial role in the pathogenesis of non-alcoholic fatty liver disease (NAFLD) [21], since this receptor is linked to dyslipidemia [22]. Given that SR-B1 is a multifunctional receptor involved in the metabolism of different lipoproteins, the purpose of this review is to highlight the association between SR-B1 and CVD risk through mice and human genetic studies.

## 2. SR-B1 in Lipoprotein Metabolism

SR-B1 is mainly identified for promoting selective uptake of CE from HDL or other lipoproteins to cells by a non-endocytic process [23,24,25,26,27,28]. Moreover, SR-B1 mediates selective hepatic uptake of HDL-CE, free cholesterol (FC), triglycerides (TG), and phospholipids by a three step mechanism [24,25,26,27]. First, cholesterol-rich donor lipoprotein particles could bind to the extracellular loop domain of the receptor. Then, SR-B1 could promote the transfer of CE from the lipoprotein particles to the plasma membrane, and finally, the cholesterol poor lipoprotein particles could release back into the circulation [3,23,24]. Furthermore, SR-B1 requires oligomerization to promote selective lipid uptake, but not HDL binding [26,27,28,29,30,31]. Although the mechanism by which SR-B1 facilitates the transfer of CE to the plasma membrane is not fully understood, a model has been proposed in which a hydrophobic tunnel is formed by the extracellular domain of the receptor between lipoprotein particles and the cell membrane through which CE diffuse in a concentration gradient manner [27,32]. The recent publication of the high-resolution crystal structure of the extracellular domain of LIMP-2, a homologue of SR-B1, supports the validity of this mechanism [33]. 

In addition to selective CE uptake, SR-B1 also facilitates the efflux of free cholesterol between cells and lipoproteins [34,35]. Briefly, this mechanism carried out by HDL is known as RCT and consists of the transport of cholesterol via HDL from peripheral tissues such as macrophages or endothelial cells to the liver for cholesterol excretion, bile acid production or steroid hormone synthesis in steroidogenic organs [26,36]. Apart from SR-B1, two more receptors are involved in this process: ATP-binding cassette A1 (ABCA1), that mediates unidirectional efflux of cholesterol and phospholipids to apolipoprotein (apo) A-I and apo E [27,37], and ATP-binding cassette G1 (ABCG1), that promotes unidirectional efflux of cholesterol to nascent HDL particles [27,38].

## 3. SR-B1, an Important Participant in the Development of Cardiovascular Disease

**Figure 1:**
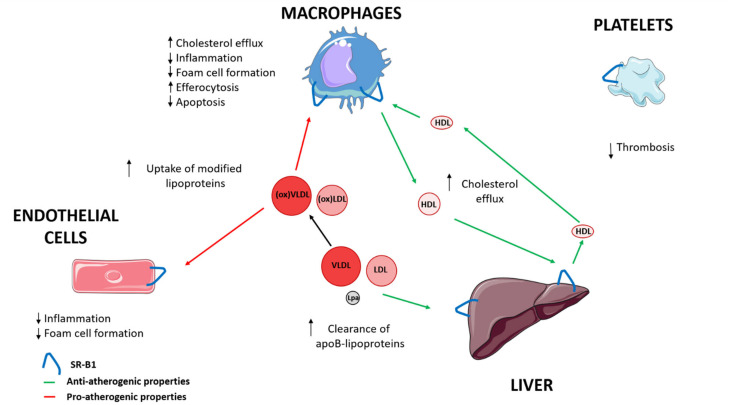
The role of SR-B1 in progression of atherosclerosis. LDL, low-density lipoprotein; Lp(a), lipoprotein (a); VLDL, very low-density lipoprotein; ↑ increase; ↓ decrease.

SR-B1 has been involved in the progression of atherosclerosis [4]. SR-B1, via HDL, contributes to the transport of cholesterol from macrophages through cholesterol efflux to the liver, and is also implicated in reducing inflammation and oxidation [3,4,27]. SR-B1 interaction with HDL modulates macrophage inflammation through activation of Akt and decreased activation of nuclear factor-κB (NF-κB), promoting the release of anti-inflammatory cytokines, including interleukin 10 and transforming growth factor-beta (TGF-ß) [26,39]. In the endothelial cells, SR-B1 inhibits inflammation via endothelial nitric oxide synthase (eNOS) activation and expression of the antioxidant enzyme, 3-beta-hydroxysteroid-delta 24-reductase (DHCR24) [4,40]. HDL and apo A-I also reduce oxidative modification of apo B containing lipoproteins [4]. Furthermore, SR-B1 in macrophages and endothelial cells could suppress the progression of atherosclerosis by modifying cholesterol trafficking and reducing atherosclerotic lesion through limiting foam cell formation [41,42]. Moreover, SR-B1 in macrophages and endothelial cells could also promote the uptake by HDL of modified lipoproteins that contribute to the development of early atherosclerotic lesions [43,44,45]. Hepatic SR-B1 mediates the clearance of VLDL, LDL, and Lp(a), whose accumulation in plasma facilitate the progression of atherosclerosis [16,17,18,19]. SR-B1 promotes the reduction of apoptosis, and mediates efferocytosis of apoptotic cells in macrophages of atherosclerotic lesions [46,47]. Platelet SR-B1 has been implicated as a negative controller in the development of thrombosis [48,49] (Figure 1) (Table 1).

## 4. Studies in Gene-Targeted Mice Related to Lipoprotein Metabolism and Cardiovascular Disease

Studies using mice models contribute to elucidate the role of SR-B1 in cholesterol homeostasis, lipoprotein metabolism and atherosclerosis, as shown by global gene deletion or overexpression of SR-B1 [4,26]. Despite the fact that the tissue expression of SR-B1 in humans is similar to that in mice [17], the metabolism of lipids and lipoproteins is different between both species [50,51]. Mice transport the bulk of plasma cholesterol in HDL, while plasma cholesterol in humans is carried predominantly by apo B containing lipoproteins such as LDL and VLDL [52]. This discrepancy between mice and humans is caused by the lack of cholesteryl ester transfer protein (CETP) in mice. This protein mediates the exchange of cholesterol and TG between HDL and LDL/VLDL. Therefore, humans have an alternative pathway for cholesterol transported by HDL to reach the liver through transferring cholesterol to LDL and VLDL and then, these lipoproteins are uptaken to the liver by the LDL receptor [41,49].

The absence of SR-B1 has been shown to accelerate the onset of atherosclerosis although SR-B1 knockout mice displayed two-fold elevated HDLc levels [53,54,55] whereas mice overexpression of SR-B1 showed decreased atherosclerosis [56,57], exhibiting the potential important role of SR-B1 in the pathophysiology of CVD. The increased atherosclerosis observed in SR-B1 knockout mice could be a consequence of reduced cholesterol efflux from macrophages to HDL and an impaired delivery of HDL-CE to the liver [54]. However, the expression of human CETP in SR-B1 deficient mice reduced HDLc plasma levels, although this genetic manipulation was not able to protect mice from atherosclerosis, suggesting that SR-B1 could have protective properties in addition to its role in the RCT mediated by HDL particles [58]. 

Studies in which global SR-B1 deletion has been carried out have demonstrated its relevance in lipoprotein metabolism [53]. Total cholesterol levels were significantly increased in SR-B1 knockout mice as well as HDLc and HDL size. This fact was a result of enhancement of the core of the HDL particles with CE and was suggested to occur due to the failure of hepatic SR-B1 to selectively uptake this core of CE [53]. As a consequence, the biliary cholesterol and adrenal cholesterol were decreased in SR-B1 deficient mice [53,55,59] and showed elevated concentrations of LDLc, VLDL cholesterol, and Lp(a) after human Lp(a) infusion [18,53]. These mice also had a modified cholesterol distribution in platelets, that altered platelet aggregation [48]. Other proatherogenic effects have been associated with the blockage of SR-B1 function, including altered transport of HDLc through the endothelial cells [3,39] and impaired reendothelialization in mouse arteries [3,60]. Moreover, previous studies have shown that female SR-B1 knockout mice were more susceptible to accelerated atherosclerosis and infertility than male mice [55]. A similar phenotype as that exhibited by SR-B1 deficient mice was found in hypomorphic liver-specific SR-B1 knockout mice [53,55,61]. Besides, Huby et al. have exposed that hypomorphic SR-B1 knockout mice, that displayed a reduce expression of SR-B1 in multiple non-hepatic tissues, needed additionally specific deletion of hepatocyte SR-B1 to increase the predisposition for the development of atherosclerotic lesions [3,62]. 

SR-B1 deficient mice in the background of apo E or LDL receptor depletion have revealed accelerated atherosclerosis and increased LDLc without significant modifications in HDLc levels, proposing that reduced LDLc clearance could play a role in the increased atherosclerosis in these mice [63,64]. SR-B1 depletion in apo E knockout mice showed severe dyslipidemia, early occlusive atherosclerotic coronary artery disease (CAD), spontaneous myocardial infarctions, severe cardiac dysfunction, and mice died prematurely between six and eight weeks of age [28,65]. Consequently, these double knockout mice might be a suitable animal model to evaluate the mechanisms involved in the development of complex CAD, myocardial infarction and heart failure, as well as for preclinical testing of potential genetic or pharmacological treatments for coronary heart disease (CHD) [55]. Interestingly, in support of the anti-atherogenic properties of hepatocyte SR-B1, liver-specific transgenic or adenoviral overexpression of SR-B1 in hyperlipidemic apo E or LDL receptor knockout mice had demonstrated the reduction in atherosclerosis predisposition [3,56,66]. 

Regarding SR-B1 in macrophages, Van Eck and collaborators investigated the role of macrophage SR-B1 in atherosclerosis by employing the bone marrow transplantation technique to particularly modulate SR-B1 expression in leukocytes in atherosclerosis-susceptible LDL receptor knockout mice [3,43]. In agreement with a pro-atherogenic role of macrophage SR-B1, the early steps involved in the development of atherosclerosis in the LDL receptor knockout mice were prevented by the particular depletion of SR-B1 function in bone marrow-derived cells [3,43]. Moreover, in normolipidemic C57BL/6 mice fed with an atherogenic cholic acid-containing diet, the deficiency of bone marrow-specific SR-B1 reduced the development of atherosclerosis [3,43]. In contrast to small macrophage-rich lesions, a notable anti-atherogenic function for macrophage SR-B1 was found in advanced phases of the disease. Transplantation of deficient SR-B1 bone marrow into lethally irradiated LDL receptor knockout mice promoted the development of greater lesions in the context of a similar extent of hyperlipidemia [3,43]. In addition, SR-B1 inactivation in bone marrow derived cells induced apoptotic cell accumulation within atherosclerotic plaques, due to reduced efferocytosis by SR-B1-deficient macrophages, increasing necrotic core development [46]. 

Galle-Treger and collaborators have also demonstrated the SR-B1 anti-atherogenic properties. In their study, they generated mice deficient for SR-B1 in monocytes/macrophages and transplanted their bone marrow into LDL receptor knockout or in mice expressing CETP. These mice exhibited accelerated aortic atherosclerosis characterized by larger macrophage-rich areas and decreased macrophage apoptosis when were fed with a cholesterol-rich diet [67]. They found that expression of apoptosis inhibitor of macrophage was induced in SR-B1-deficient macrophages; therefore, they suggested that macrophage SR-B1 was involved in plaque growth by controlling macrophage apoptosis [67]. These results contrast with those obtained by Tao et al. reporting increased apoptotic cell accumulation using the same experimental approach [46]. These contradictions might show that macrophage-SR-B1 exerted various protective activities that depended on the lesion stage development [46,67]. In this sense, the shift from SR-B1 as a pro-atherogenic factor to an anti-atherogenic factor seemed to take place at a lesion size between 150.000 and 250.000 mm 2 [3]. Furthermore, LDL receptor knockout mice that received bone marrow from mice deficient in ABCA1 and SR-B1 exhibited a noticeable increase in the extent of atherosclerosis as compared to ABCA1 deficiency bone marrow in LDL receptor knockout recipient mice [3,68]. These studies showed that the role of SR-B1 in macrophages within atherosclerotic lesions appeared to be dependent on the lipid context and the stage on atherosclerosis development. Therefore, to better evaluate the function of macrophage SR-B1 in human atherosclerosis, mice models with a more human-like lipoprotein profile would be needed [3].

Opposite effects than those shown in SR-B1 deficient mice were found in hepatic overexpression of SR-B1 in mice. These mice displayed lower levels of circulating HDLc, increased HDL-CE clearance and transport of cholesterol from the liver into the bile, and increased biliary cholesterol content [56,57,59,60]. Hepatic overexpression of SR-B1 was associated with reduced concentrations of VLDL and LDL [57,61] as well as increased plasma clearance of Lp(a) [18]. Arai et al. have proposed that at least a part of the protection against atherosclerosis related to SR-B1 overexpression could be attributed to its role on apo B containing lipoproteins such as LDL and VLDL [66]. Studies developed by Vaisman and collaborators have presented that endothelial cell-specific overexpression of SR-B1 in high fat/high cholesterol diet-fed C57BL/6 mice and apo E knockout mice decreased atherosclerosis susceptibility [3,69].

In summary, mice studies have showed the relevant involvement of SR-B1 in the development of CVD. In addition, mice models are needed in order to evaluate the diverse roles of SR-B1 in the different cells and tissues to develop new SR-B1-target treatments for CVD. However, mice experiments have some limitations due to the differences in lipoprotein metabolism between mice and humans. 

## 5. Human Genetic Variants of *SCARB1* in Lipoprotein Metabolism and Cardiovascular Disease

**Figure 2:**
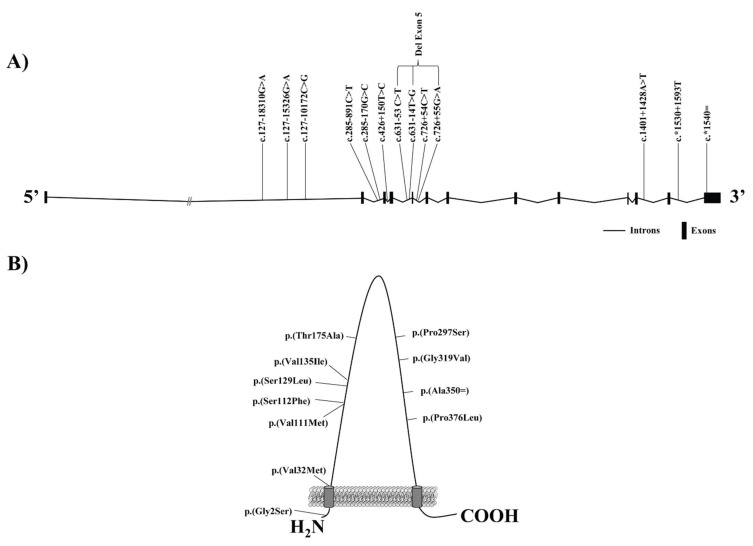
Localization of intronic (**A**) and exonic (**B**) variants involved in lipoprotein metabolism described in this review are shown in a schematic *SCARB1* gene (**A**) and SR-B1 protein (**B**).

The evaluation of gene variants in *SCARB1* has contributed to elucidate the association of SR-B1 in the regulation of lipoprotein metabolism in humans (Table 2) (Figure 2). The first study to show the involvement of genetic variants at *SCARB1* on SR-B1 in humans described three common polymorphisms, at exons 1 and 8 and intron 5 in Spanish Caucasians (Table 2) (Figure 2) [70]. The single nucleotide variant (SNV) in exon 1 at base pair (bp) 4 encoded a change from glycine to serine at the second aa position, c.4G>A, p.(Gly2Ser). At exon 8, the SNV was confirmed to compromise a change in bp 1050 encoding aa 350 (c.1050T > G, p.(Ala350 = )), but there was no aa change. Finally, SNV close to exon 5 was located in adjacent intron but was not in the canonical splice-site sequence (c.726 + 54C > T). Subsequent analyses demonstrated the involvement of SR-B1 variants in lipoprotein metabolism. In this sense, exon 1 variant (c.4G > A, p.(Gly2Ser)) was significantly related to an increase of HDLc and lower LDLc levels in men linked with a reduced atherogenic profile. Women carriers of exon 8 genetic variant exhibited reduced LDLc values. Intron 5 variant was related to higher body mass index in women and lower TG levels in men [70]. Further population-base studies of *SCARB1* gene polymorphisms established the involvement of these genetic variants in plasma lipoprotein profile [71,72], although several studies resulted in conflicting results [73,74] or failed to find an association [75,76]. These studies focused on different populations, including women and estrogen therapy [71], diabetics [72,75], different ethnic groups [77,78,79,80] individuals with familial hypercholesterolemia [51,81], and CVD patients [73,74,75,76,77,78,79,80,81,82,83]. Moreover, additional factors related to lipoprotein metabolism were evaluated in association with these genetic variants including the response to different diets [84,85] and pharmacological interventions [86,87]. The differences found in lipoprotein metabolism between studies is possible to be explained by sample size, gender, ethnic groups, physical condition, and other variables [87]. The precise role of *SCARB1* genetic variants in the alterations observed in lipoprotein metabolism is unknown [51]. The polymorphism located in exon 1, p.(Gly2Ser), results in an aa change in the protein. Further experiments showed that selective cholesterol uptake resided in the extracellular domain of SR-B1 receptor, but the aa change takes place in the intracellular N-terminus [51,71]. Although exon 8 polymorphism does not involve an aa change, it has been shown that it decreases protein expression by changing RNA secondary structure, therefore could alter the functionality of the receptor [88,89]. Intron 5 SNV has no recognized effect on splicing or gene expression [71]. Several studies have suggested that none of these three polymorphisms is functional and that the associations found are a consequence of linkage to other genetic variants at the SR-B1 locus or neighboring loci yet to be recognized [51,87].

In humans, more genetic variants in *SCARB1* have been reported (Table 2) (Figure 2). A study developed in the Taiwanese Chinese population found two novel variants in the *SCARB1* gene promoter region. One of them was an 11-bp CCCCGCCCCGT deletion from positions −140 to −150 (c.-140_-150del) from the transcription start site, corresponding to a Sp1 binding site, and the other one was a C→ to T substitution at position −142 (c.-142C>T). In vitro experiments showed that the promoter containing the −140 to −150del allele exhibited reduced activity and this finding was in accordance with the higher plasma HDLc levels presented in these subjects. However, no significant results were identified in the c.-142C>T [77]. Research developed in the Amish population showed that the missense variant in exon 3, c.403G>A, p.(Val135Ile) was associated with higher levels of HDLc in women [72]. This genetic variant was also related to higher apo B levels in a study developed in US non-hispanic white people with extreme values of HDLc [80]. The objective of the study carried out by Niemsiri et al. was to resequence the *SCARB1* gene in selected US non-Hispanic white individuals with extreme HDLc levels to identify common and rare variants and then to evaluate the role of the identified variants with plasma HDLc, LDLc, TG, and apo B levels [80]. Single-locus analysis revealed three nominally significant associations with HDLc: c.127–18310G>A, c.*1530+1593T, and c.*1540=, that have been reported to be genome-wide significant [80,93]. In addition, three common *SCARB1* polymorphisms (c.285–891C>T, c.285–170G>C, and c.426+150T>C) showed significant associations with apo B [80]. Notably, c.285–891C>T has been previously established to be related to carotid intima-media thickness and incidence of CHD [90]. The risk of allele c.285–891C>T has also been found to be associated with lower SR-B1 protein levels [91]. 

Naj et al. found that the c.127–15329G>A genetic variant was associated with higher common carotid intima-media thickness, one of the subclinical atherosclerosis features, in multiple ethnic groups [90]. Chiba-Falek et al. showed that c.1401+1428A>T was associated with endogenous estradiol levels, HDLc, TG, and the TG:HDLc ratio in postmenopausal Caucasian women. In addition, this study found that this variant was related to decreased level of liver SR-B1 in women under 45 years old, suggesting that this SNV could be associated with CHD [94]. However, no associations of these variants were found in the Han Chinese population [92]. Naj et al. also showed that the C allele of c.127–10172C>G variant was related to higher common carotid intima-media thickness in African American, Chinese, European American, and Hispanic subjects, although, this association was independent of lipid levels [90]. However, this genetic variant in the Han Chinese population seemed to increase CHD risk as well as the HDLc level [92].

Vergeer and collaborators sequenced the gene encoding SR-B1 in the Caucasian population with high HDLc levels and reported a family with a mutation in the nucleotide 889 producing a proline to serine substitution at aa position 297, c.889C>T, p.(Pro297Ser). This aa change was located at the extracellular loop, suggesting it to be functionally relevant [98,99]. These subjects displayed increased HDLc, reduced capacity to efflux cholesterol from macrophages, impaired platelet function, and decreased adrenal steroidogenesis. In addition, primary murine hepatocytes expressing SR-B1 p.(Pro297Ser) exhibited reduced cholesterol uptake from HDL. However, carotid intima-media thickness was similar in carriers and family controls. Vergeer et al. explained that the statistical power to distinguish a difference in carotid intima-media thickness was low, assumed the small number of carriers and their relatively young age [41]. Further experiments found that p.(Pro297Ser) mutation alters the protein composition of HDL and LDL/VLDL [99]. Applying the same strategy explained above, Brunham et al. identified two novel missense mutations, c.335C>T, *p*.(Ser112Phe), and c.523A>G, *p*.(Thr175Ala) by sequencing *SCARB1* gene in the Caucasian ancestry population with elevated HDLc level. p.(Ser112Phe) and *p*.(Thr175Ala) mutations occurring in the large extracellular loop of the SR-B1 protein. None of those mutation carriers had a history of CVD [98]. In vitro studies showed that both mutant receptors exhibited altered HDL binding, selective uptake of HDL-CE, and delivery of FC from cells to HDL. These results suggest that increased plasma HDLc in these settings could not be associated with reduced risk of CVD [100].

As mentioned before, Yang and collaborators described, by experiments in vitro and in vivo, the role of Lp(a) as ligand of SR-B1, which mediates the selective uptake of Lp(a)-associated lipids [18]. To assess this new mechanism in humans, two cohorts, that included multi-ethnic populations, were examined for combined elevations of HDLc and Lp(a) to evaluate its interaction with *SCARB1* genetic variants and with reduced function of SR-B1 protein [89]. Five novel missense or splice site variants in *SCARB1* were identified. One SNV in exon 3 results in a missense serine to leucine substitution at aa 129 (c386C>T, p.(Ser129Leu)). Two mutations produce the deletion of exon 5: the splicing variant c.631–14T>G and a compound mutation at c.631–53C>T and c.726+55G>A. In the SR-B1 isoform 2, c.1495G>A induces a p.(Gly499Arg) substitution in the carboxy-terminal tail. Finally, a missense substitution at exon 3 results in aa change in the extracellular loop of SR-B1: p.(Glu130Gly) (c.386A>G). The function of p.(Gly499Arg) and *p*.(Glu130Gly) was not tested. In addition, they also distinguished two common polymorphisms described before, p.(Gly2Ser) and *p*.(Ala350A=). Posterior in vitro studies showed that the *p*.(Ser129Leu) polymorphism and the mutations that caused the deletion of exon 5 lower CE uptake from HDL and Lp(a) [89]. The consequence of high HDLc/high Lp(a) phenotype on human CVD is not established. In this sense, Lp(a) is a pro-atherogenic lipoprotein [19,89], whereas high HDLc could reduce CVD risk [5,6]; however, not all conditions that elevate HDL are protective in humans [95,96].

Recently, Zanoni et al. identified a missense mutation in *SCARB1*, which replaces proline at the 376 position by leucine (c.1127C>T, p.(Pro376Leu)), by targeted sequencing of coding regions of lipid-modifying genes in individuals of European ancestry with extremely elevated HDLc levels. This aa substitution occurs on the extracellular loop proximal to the C-terminal transmembrane domain. In the homozygous subject, p.(Pro376Leu) mutation altered posttranslational processing of SR-B1, abolished selective HDLc uptake in vitro and in vivo experiments and reduced the carotid intima-media thickness. In addition, a meta-analysis of 16 studies showed that p.(Pro376Leu) carriers had a significantly higher risk of CAD compared with non-carriers [95]. In agreement with these results, Samadi et al. concluded that carriers of p.(Pro376Leu) mutation were more susceptible to develop CVD, although no differences were found in HDLc levels;serum HDL lipid peroxidation, measured as dysfunctional HDL, was increased in the presence of *p*.(Pro376Leu) mutation [96]. This study was developed in CVD patients from the Mashhad Stroke and Heart Atherosclerotic Disorders (MASHAD) cohort. Some authors have suggested that given the extremely low and variable carrier rate of this genetic variant between study groups in the meta-analysis and taking into account that this study was relatively specific to Ashkenazi Jews, *p*.(Pro376Leu) mutation could be an indirect indicator for a substratum of the population [8,101]. Subsequent to the publication of Zanoni and collaborators’ results, Helgadottir et al. decided to study the hypothesis that alleles in *SCARB1* gene related to higher levels of HDLc are also associated with increased risk of CAD in the relatively homogeneous population of Iceland. They identified three novel *SCARB1* missense genetic variants, c.956G>T, p.(Gly319Val), c.331G>A, p.(Val111Met), and c.94G>A, p.(Val32Met), associated with increased HDLc levels. p.(Gly319Val) and p.(Val111Met) variants take place in the large extracellular loop of the SR-B1 protein. Moreover, they also identified a missense variant, p.(Val135Ile) described before in Amish population and in US non-Hispanic white people [72,80]. They studied the association between the described polymorphisms in Iceland population and CAD risk, although no linkage was found. The lack of relation between these polymorphisms producing high HDLc levels and the risk of CAD suggested no alteration in the hepatocellular trafficking of cholesterol to bile. However, they proposed that an enhancement of CETP-mediated exchange of CE from HDL to apo B containing lipoproteins could prevent the increase in HDLc in genetically impairment of SR-B1. Therefore, no effect was found on the hepatic cholesterol clearance in carriers of the Icelandic variants. To validate this hypothesis, they evaluated the formation of gallstone since it has been described as a manifestation of cholesterol hypersecretion to bile [8,102]. They found a significant increase of gallstone formation in p.(Val111Met) carriers, supporting the finding that an increase of HDLc levels in humans impairs cholesterol excretion through bile [8]. May et al. described a patient from the Lipid Genetics Clinic at the London Health Sciences Centre, University Hospital (London, ON, Canada) who had elevated levels of HDLc. This subject was found to carry a heterozygous variant of *SCARB1* consisting in a missense arginine to cysteine substitution at aa 174 (p.Arg174Cys). This mutation showed reduced cholesterol transport, suggesting an impairment in the cholesterol clearance [97]. 

*SCARB1* genetic variants research and the description of mutations in human *SCARB1* have established the involvement of SR-B1 in regulating lipoprotein metabolism in humans [27]. However, based on the divergent results, more research is needed in order to elucidate the role of SR-B1 variants in the increment of HDLc levels and CVD risk.

## 6. Genome-Wide Association Studies of *SCARB1*

Genome-wide association studies (GWAS) have been used to distinguish disease susceptibility loci, proposing novel genetic variants at new loci related to CVD and serum HDLc concentrations [103]. GWAS for plasma lipids first associated *SCARB1* common polymorphisms with HDLc in 2010 [93]. In this study, the principal SNV in *SCARB1* implicated from this GWAS, c.*1540G>T, was related to increased HDLc [93]. However, the relationship of the haplotype to *SCARB1* expression has not been confirmed yet at molecular level [104]. Recently, two studies identified significant associations for the *SCARB1* locus with CAD risk [103,104]. Remarkably, these studies did not find that this CAD-associated haplotype is in linkage disequilibrium with the HDLc associated haplotype from GWAS for lipids. However, Webb and collaborators showed that the lead variant, c.127–4800C>T, has a strong association with plasma LDLc and TG levels, and this genetic variant has also been related to increased lipoprotein-associated phospholipase A2 activity [103]. In addition, c.127–14490G>A is related to expression of *SCARB1* in the intestine [103]. The causal mechanisms explaining the apparent lack of association between HDLc, CAD traits and *SCARB1* significant signals continue to be unknown, suggesting that further functional genomic investigations to better comprehend the function of these regulatory variants are needed [105].

## 7. Conclusions

SR-B1 not only plays a role in the metabolism of HDL but also is involved in the clearance of LDL, VLDL, and Lp(a), proposing an important participation in the development of CVD [16,17,18]. Therefore, mice and human genetic studies have shown genetic variants in *SCARB1* gene-induced higher levels of these mentioned lipoproteins and can alter their composition. In addition, SR-B1 variants can modify cholesterol efflux of macrophages and hepatic reverse cholesterol transport, reduce adrenal steroidogenesis, alter platelet functions, as well as decrease the selective uptake of CE. 

The available data propose that plasma HDLc levels are not an optimal therapeutic target and that the quality of the HDL particles circulating in plasma could be more critical for therapeutic interventions than HDLc concentration [14], since high HDLc levels may not always be protective against CVD [95,106]. In fact, European Society of Cardiology and European Atherosclerosis Society Guidelines for the Management of Dyslipidaemias [107] do not recommend HDLc as a target for treatment. The differential HDLc uptake by SR-B1 among subjects may be one factor associated with modified HDL quality and functionality associated with CVD risk without substantial modification of HDLc concentration. 

The contradictory results in human SR-B1 variants in the relationship between increased HDLc levels and CVD risk, as well as GWAS of *SCARB1*, suggest the involvement of other pathways. Furthermore, human SR-B1 variants provide evidence that modulating different functions of SR-B1 might improve the development of CVD [8]. It is important to mention that the role of SR-B1 in CVD is cell/tissue type-specific [3,4,44], highlighting the complexities of potential therapeutic development with SR-B1 modulating agents [8]. Therefore, further research is needed in order to develop a proper SR-B1-based therapeutic approach.

## Figures and Tables

**Table 1:** The role of SR-B1 in atherosclerosis. LDL, low-density lipoprotein; Lp(a), lipoprotein (a); VLDL, very low-density lipoprotein; ↑ increase; ↓ decrease.

	Anti-Atherogenic Properties	Pro-Atherogenic Properties
**Liver**	↑ Cholesterol efflux [16,17,18,19]	
	↑ Clearance LDL, VLDL and Lp(a) [16,17,18,19]	
**Macrophages**	↑ Cholesterol efflux [16,17,18,19]	↑ Uptake modified lipoproteins [43,44,45]
	↓ Inflammation [39]	
	↓ Foam cell formation [40]	
	↑ Efferocytosis [47]	
	↓ Apoptosis [46]	
**Endothelial cells**	↓ Inflammation [40]	↑ Uptake modified lipoproteins [43,44,45]
	↓ Foam cell formation [41]	
**Platelets**	↓ Thrombosis [48,49]	

**Table 2:** Effects of *SCARB1* gene variants on serum lipid profile. cDNA position was related to the *SCARB1* gene (NM.005505.5; encoding SR-B1). † Nucleotide position 1 is the first nucleotide at the ATG initiation codon. * Acton et al., 1999 results are presented in this table since it was the first time that these variants were described, although several studies have shown different results. NA indicates not applicable. Apo, apolipoprotein; CE, cholesterol ester; CHD, coronary heart disease; CVD, cardiovascular disease; HDL, high-density lipoprotein; HDLc, high-density lipoprotein cholesterol; LDL, low-density lipoprotein; LDLc, low-density lipoprotein cholesterol; Lp(a), lipoprotein (a); SR-B1, scavenger receptor B class 1; VLDL, very low-density lipoprotein; ↓ decrease; ↑ increase.

DNA Variant †	Protein Variant	Study Subjects	rsID	Exon/Intron	Variant Effect	Reference
c.4G > A	p.(Gly2Ser)	Spanish Caucasians	4238001	Exon 1	↑ HDLc in men	Acton et al., 1999 * [70]
					↓ LDLc levels in men	
c.1050T > G	p.(Ala350Ala)		5888	Exon 8	↓ LDLc values in women	
4c.795 + 54C > T	NA		NA	Intron 5	↑ body mass index in women	
					↓ TG levels in men	
c.-140_-150del	NA	Taiwanese Chinese population	NA	NA	↑ levels of HDLc	Hsu et al., 2003 [77]
					↓ promotor activity (in vitro)	
c.403G > A	p.(Val135Ile)	Amish population	5891	Exon 3	↑ levels of HDLc in women	Roberts et al., 2007 [72]
		US non-Hispanic white with extreme HDL-C level			↑ apo B levels	Niemsiri et al., 2014 [79]
c.127-18310G > A	NA	US non-Hispanic white with extreme HDL-C level	11057844	Intron 1	Associated to HDLc	Niemsiri et al., 2014 [80]
c.*1530 + 1593T	NA		701106	Intron 12		
c.*1540 =	NA		838880	3 prime UTR		
c.285–891C > T	NA	US non-Hispanic white with extreme HDL-C level	2343394	Intron 2	↑apo B levels	Niemsiri et al., 2014 [80]
		Multiethnic groups			Associated with carotid intima-media thickness	Naj et al., 2010 [90]
					Related to CHD	
					↓ *SCARB1* protein levels	West et al., 2009 [91]
c.127–15326G > A	NA	Multiethnic groups	10744182	Intron 1	↑ common carotid intima-media thickness	Naj et al., 2010 [90]
		Hen Chinese population			No association	Zeng et al., 2017 [92]
c.127–10172C > G	NA	Multiethnic groups	10846744	Intron 1	↑ common carotid intima-media thickness independent of lipid levels	Naj et al., 2010 [93]
		Hen Chinese population			↑ HDLc levels	Zeng et al., 2017 [92]
					↑ CHD risk	
c.1401 + 1428A > T	NA	The suburban community of Rancho Bernardo	838893	Intron 11	Related to endogenous estradiol levels, HDLc, TG, and the ratio TG:HDLc in postmenopausal Caucasian women	Chiba-Falek et al., 2010 [94]
					Associated with ↓level of liver SR-B1 in women under the age of 45	
		Hen Chinese population			No association	Zeng et al., 2017 [92]
c.889C > T	*p*.(Pro297Ser)	Caucasian population with HDLc above the 95th percentile (Netherlands)	387906791	Exon 7	↑ HDLc levels	Vergeer et al., 2011 [41]
					↓ Cholesterol efflux macrophages (in vitro)	
					↓ adrenal steroigenesis (in vitro)	
					Changes in platelet function (in vitro)	
					No alterations in carotid intima-media thickness	
					Changes in HDL, LDL and VLDL composition	
c.335C > T	*p*.(Ser112Phe)	Caucasian ancestry population with high levels of HDLc	397514572	Exon 3	Alterations in HDL binding (in vitro)	Brunham et al., 2011 [7]
c.523A > G	*p*.(Thr175Ala)		187831231	Exon 4	Modifications in selective uptake of HDL-CE (in vitro)	
					Changes in the delivery of FC from cells to HDL (in vitro)	
c.386C > T	*p*.(Ser129Leu)	Multiethnic population with high HDLc and high Lp(a)	150222965	Exon 3	↓CE uptake from HDL and Lp(a) (in vitro)	Yang et al., 2016 [89]
c.631–14T > G	delExon5		113910315	Intron 4		
c.631–53 C > T c.726+55 G > A	delExon5		77740046 59809936	Introns 4,5		
c.1127 C > T	*p*.(Pro376Leu)	European ancestry with extremely elevated HDLc levels (Ashkenazi Jews)	74830677	Exon 8	Alteration in posttranscriptional processing of SR-B1 (in vitro)	Zanoni et al., 2016 [95]
					Abolishment of selective uptake of HDL-CE (in vitro and in vivo)	
					↑ CVD risk	
		CVD patients from MASHAD cohort			No differences found in HDLc levels	Samadi et al., 2019 [96]
					HDL lipid peroxidation	
c.956G > T	*p*.(Gly319Val)	Homogenous population of Iceland	150728540	Exon 7	↑ HDLc levels	Helgadottir et al., 2018 [8]
c.331G > A	*p*.(Val111Met)		5890	Exon 3	↓ hepatic reverse cholesterol	
c.94G > A	*p*.(Val32Met)		771247110	Exon 1	No ↑ CVD risk	
c.520C > T	*p*.(Arg174Cys)	Patients with extreme levels of HDLc (Canada)	367669186	Exon 4	↓ Cholesterol transport	May et al., 2021 [97]

## Abbreviations

Aa: amino acid; ABCA1, ATP-binding cassette A1; apo, apolipoprotein; ATP-binding cassette G1 (ABCG1); bp, base pair; CAD, coronary artery disease; CE, cholesterol ester; CETP, cholesteryl ester transfer protein; CHD, Coronary heart disease; CVD, cardiovascular disease; DHCR24, 3-beta-hydroxysteroid-delta 24-reductase; eNOS, endothelial nitric oxide synthase; FC, free cholesterol; GWAS, Genome-wide association studies; HDL, high-density lipoprotein; HDLc, high-density lipoprotein cholesterol; LDL, low-density lipoprotein; LDLc, low-density lipoprotein cholesterol; LIMP-2, lysosomal integral membrane protein 2; Lp(a), Lipoprotein (a); MASHAD, Mashhad-Stroke and Heart-Atherosclerotic-Disorders; NAFLD, non-alcoholic fatty liver disease; NF-κB, nuclear factor-κB; PDZK1, PDZ Domain Containing 1; RCT, reverse cholesterol transport; SNV, single nucleotide variant; SR-B1, scavenger receptor B class 1; SR-B2, scavenger receptor B class 2; TG, triglycerides; TGF-ß, transforming growth factor-beta; VLDL, very low-density lipoprotein.
